# The Effects of Added Cellulases and Pectinases on Ruminal Fermentation Parameters and Bacterial Communities in Goats Supplemented with Macadamia Integrifolia Husks: An In Vitro Study

**DOI:** 10.3390/ani15223337

**Published:** 2025-11-19

**Authors:** Faguo Cai, Jiancheng Han, Donghong Zhu, Ximei Song, Hui Zeng, Xiaosong Zhang, Anmiao Chen, Zehua Li, Shiyang Huang, Jingbo Liu, Mao Li, Hu Liu, Hanlin Zhou

**Affiliations:** 1 Zhanjiang Experimental Station, Chinese Academy of Tropical Agricultural Sciences, Zhanjiang 524013, China; caifg9702@126.com (F.C.); hanjiancheng810@163.com (J.H.); cam1835287831@163.com (A.C.); 15309090845@163.com (Z.L.); 2 Bosar Biotechnology Research Co., Ltd., Kunming 650000, China; 3 Agricultural Products Processing Research Institute, Chinese Academy of Tropical Agricultural Sciences, Zhanjiang 524013, China; 4 Key Laboratory of Hainan Province for Postharvest Physiology and Technology of Tropical Horticultural Products, China South Subtropical Crops Research Institute, Chinese Academy of Tropical Agricultural Sciences, Zhanjiang 524091, China; ximeisongjiayou@126.com (X.S.); 13692441670@163.com (H.Z.); 5 College of Veterinary Medicine, China Agricultural University, Beijing 100193, China; zhangxsgs@163.com; 6 College of Animal Science and Technology, Guangxi University, Nanning 530004, China; yangdeng419@163.com; 7 College of Life Sciences and Agri-Forestry, Southwest University of Science and Technology, Mianyang 621010, China; jingboliu@swust.edu.cn; 8 Tropical Crops Genetic Resources Institute, Chinese Academy of Tropical Agricultural Sciences, Haikou 571101, China; limaohn@163.com

**Keywords:** Macadamia integrifolia husk, cellulases, pectinase, in vitro, goats

## Abstract

Macadamia integrifolia husk has great potential for use as a feedstuff in ruminants. However, little information regarding its effects on ruminal fermentation traits is available at present. Hence, this in vitro study aims to investigate the effects of Macadamia integrifolia husk as a substrate, supplemented with enzymes at different levels (0 g/kg cellulases + pectinases, CON; 0.5 cellulases + 0.5 g/kg pectinases, TRE1; 1.0 g/kg cellulases + 0.5 g/kg pectinases, TRE2; 1.5 cellulases + 0.5 g/kg pectinases, TRE3), on gas production, fermentation traits, and bacterial communities in goats. The results demonstrate that gas production was increased in TRE1 and TRE3 groups compared to the CON group at 6, 9, 12, 24, and 48 h (*p* < 0.05); DMD was the highest in the TRE3 group and the lowest in the CON group at 6, 12, 24, and 48 h (*p* < 0.05); and NDFD was the highest in the TRE1 group and the lowest in the CON group at 6 h, then the highest in the TRE3 group and the lowest in the CON group at 12, 24, and 48 h (*p* < 0.05). The concentrations of MCP, TVFAs, acetate, and propionate were the highest in the TRE3 group and the lowest in the CON group (*p*< 0.05). The abundance of Bacillota was the highest in the TRE1 group and the lowest in the TRE2 group (*p* < 0.05), whereas that of Synergistota and Actinomycetota was the lowest in the CON group and the highest in the TRE2 group (*p* < 0.05). Acidobacteriota was the most abundant in the TRE3 group and the least abundant in the TRE1 group (*p* < 0.05). The most abundant genus was *norank_p_Bacteroidota*, comprising approximately 29.3%, 30.6%, 30.4%, and 31.7% of the total bacteria in the CON, TRE1, TRE2, and TRE3 groups, respectively; this was followed by Succiniclasticum, comprising approximately 6.3%, 5.8%, 6.1%, and 7.0% of the total bacteria in the CON, TRE1, TRE2, and TRE3 groups, respectively. This study provides new insights regarding the use of Macadamia integrifolia husk as a feedstuff in goats; in particular, supplementation with cellulases and pectinases could effectively improve its utilization. The results suggest that the optimal supplementary levels of cellulases and pectinases are 1.5 g/kg and 0.5 g/kg, respectively.

## Simple Summary

Macadamia integrifolia husk constitutes 40% of the fresh fruit’s weight and has great prospects for use as a feedstuff for ruminants. This in vitro study aimed to explore the effects of Macadamia integrifolia husk supplemented with cellulases and pectinases on rumen fermentation parameters and bacterial communities in goats. The results reveal that cellulases and pectinases, supplemented at 1.5 g/kg and 0.5 g/kg, respectively, improved the fermentation parameters associated with Macadamia integrifolia husk.

## 1. Introduction

Macadamia integrifolia, belonging to the Proteaceae family, is a handsome evergreen tree native to eastern Australia. Its nuts are enriched in essential amino acids and polyunsaturated fatty acids, which are beneficial for human health [1,2]. Its introduction from Australia into China began in the 1970s, where it has been successfully cultivated and commercialized as a nut crop. With the cultivated area of macadamia in China exceeding 359,400 hectares in 2023, it has become the largest and fastest growing macadamia plantation country in the world, hosting 2/3 of the world’s planted area [3,4]. In China, it has been estimated that the yield of Macadamia integrifolia was 16,900 ton [4]. There are a series of by-products associated with the processing of Macadamia integrifolia nut; for example, Macadamia husks—which comprise 40% of the fresh fruit’s weight—are the outer coating of the nut-in-shell and are produced as part of the de-husking process [5]. Most of these husks end up in landfill, while few farmers use the husks to feed animals.

At present, to reduce the increasing competition between feed and food, agro-industrial by-products are being considered widely for use as feedstuffs for animal nutrition [6,7]. This is particularly relevant for herbivores due to the strong fermentation ability of their gastrointestinal tract, allowing for the digestion of fibrous biomasses which are not edible for humans. However, the overall utilization of agro-industrial by-products remains low. There are many ways to solve this problem; for example, by adding enzymes and/or probiotics, or fermentation via silage [8,9]. An in vitro study reported that adding different doses of fibrolytic enzymes to three low-quality tropical forages improved their digestibility, contributing to the sustainable intensification of livestock production in tropical countries [10]. Therefore, the addition of exogenous fibrolytic enzymes can be a sustainable way to enhance the digestibility of fiber sources in herbivore systems, based on the fact they are produced by microorganisms.

A previous in vitro study reported that the dietary inclusion of between 14% and 21% of nut skins (e.g., almond, hazelnut, or pistachio skin) might be beneficial [11]. In addition, an in vivo study reported that the simultaneous inclusion of linseed and hazelnut skin can be a profitable strategy for enriching the intramuscular fat of lambs with health-promoting fatty acids, without any adverse effects on ruminal fermentation or animal performance [12].

On this basis, we investigated the supplementation of Macadamia integrifolia husk with cellulases and pectinases through an in vitro study. The aim of this study was to provide data on the use of Macadamia integrifolia husk as a feedstuff in animal nutrition, and to verify the optimal doses of the considered enzymes to enhance rumen fermentation parameters in goats.

## 2. Materials and Methods

The procedures and analysis of this experiment were carried out at the Zhanjiang Experimental Station, Chinese Academy of Tropical Agricultural Sciences (CATAS). All procedures involving animal use were previously approved by the Animal Ethics Committee of Zhanjiang Experimental Station (protocol no. CATAS-20250005ZES; approval date: 9 April 2025).

### 2.1. Macadamia Integrifolia Husk Sample Collection

The Macadamia integrifolia husk was collected from the Germplasm Resource Nursery (China 21°09′52″ N and 110°16′24″ E) belonging to the Institute of South Subtropical Crops, CATAS. A total of 5 kg of Macadamia integrifolia husk from the same species (Nanya No. 12) was collected in September of 2024 and then dried in oven at 65 °C to a constant weight. Finally, the Macadamia integrifolia husk was ground, passed through 1 mm sieves, and stored at 4 °C until chemical measurements and the in vitro study were performed. 

### 2.2. Experimental Design and Substrate Preparation

The substrate of Macadamia integrifolia husk was supplemented with cellulases and pectinases at varying levels: only Macadamia integrifolia husk (CON), Macadamia integrifolia husk with 0.5 g/kg cellulases and 0.5 g/kg pectinases (TRE1), Macadamia integrifolia husk with 1.0 g/kg cellulases and 0.5 g/kg pectinases (TRE2), and Macadamia integrifolia husk with 1.5 g/kg cellulases and 0.5 g/kg pectinases (TRE3). About 0.4 g of substrate were placed into individual nylon bags (4.5 cm × 5 cm) and 6.0 g beads were added to these bags to ensure that the samples could sink in the rumen inoculum. The cellulases and pectinases were provided by Bosar Biotechnology Research Co., Ltd. (Kunming, China).

### 2.3. In Vitro Experimental and Sample Collected

Six healthy goats with similar body weight (14.20 ± 0.25 kg) were selected as rumen fluid donors. These donors were fed a total mixed ratio with a roughage and concentrate mixture (1:1) and had ad libitum access to fresh water and mineral mixture. Ruminal liquid was collected by using an oral stomach tube before the morning feeding from each animal, which was then filtered through 4 layers of cheesecloth. The procedure of in vitro incubation followed that outlined previously by Liu et al. [13], using a 100 mL Menke fermenter (Model Fortuna, Haberle Labortechnik, Lonsee, Germany) placed in a shaking water bath maintained at 39 °C. Briefly, 200 mL volumes of rumen fluid from each goat were combined and mixed at a ratio of 1:2 (*v*/*v*) with a reduced buffer medium under CO_2_ flushing. The inoculum was prepared according to Liu et al. [13]. Three standard hay samples (produced by University of Hohenheim, Germany) and three blanks with only buffered rumen fluid were incubated concomitantly for gas correction. There were 8 replicates per treatment, obtained at each of the following time points: 6, 12, 24, and 48 h; in particular, the experiment consisted of two runs with four replicates per treatment per time point. Gas production was recorded according to the fermenter volume after 0, 3, 6, 9, 12, 24, and 48 h. The gas was emptied when the volume of gas produced and buffered rumen fluid exceeded 100 mL. Incubation was stopped at 6, 12, 24, and 48 h, and the filter bags with substrate residues from each Menke fermenter were placed on ice water (temperature at 0 °C) to terminate fermentation, then later used for digestibility measurements. At 48 h, the pH was determined immediately using a portable pH meter. Each fermentation fluid sample was collected at 48 h and divided into 4 tubes. Then, 4 mL of fermentation fluid was mixed with 4 mL de-proteinizing solution (100 g/L metaphosphoric acid and 0.6 g/L croconic acid) for volatile fatty acid (VFA) analysis; 4 mL of fermentation fluid was mixed with 4 mL hydrochloric acid solution (0.5 mmol/L) for ammonia-N and microbial protein (MCP) measurement; 5 mL of fermentation fluid was collected from each fermenter for bacterial DNA extraction; and the rest was stored in 50 mL centrifuge tubes. All the fermentation fluid samples were kept at −80 °C, with the initial (0 h) sample used to calculate the net yield of total VFAs.

### 2.4. Sample Analysis

For the substrates of Macadamia integrifolia husk, we measured the dry matter (method 942.45), crude protein (method 976.05), organic matter (method 942.05), and ether extract (method 920.29), according to the Association of Official Analytical Chemists [14]. The neutral detergent fiber and acid detergent fiber were determined using an automatic fiber analyzer (Ankom Technology, Fairport, NY, USA), according to Van Soest et al. and Robertson and Van Soest, respectively [15,16]. The dry matter digestibility (DMD), neutral detergent fiber digestibility (NDFD), and acid detergent fiber digestibility (ADFD) values were calculated from their initial contents in the substrate at 0 h and after 6, 12, 24, and 48 h of fermentation. The chemical composition of the Macadamia integrifolia husk showed in Table 1.

The frozen fermentation fluid from 48 h was thawed at 4 °C and centrifuged at 3770× *g* for 15 min at 4 °C. Concentrations of VFAs—including acetate, propionate, butyrate, isobutyrate, valerate, and isovalerate—were determined. The supernatant (1.5 mL) was placed in 2.0 mL tubes with 0.2 mL 2-ethyl butyric acid as an internal standard. After stirring and then standing for 30 min, the mixture was centrifuged again at 3770× *g* for 15 min. The supernatant was filtered (0.22 μm) for determination of VFAs using a gas chromatograph (SP-3420A, Beifen-Ruili Analytical Instrument Co., Ltd., Beijing, China) equipped with an AT-FFAP type capillary column (30 m × 0.32 mm × 0.5 μm). The Ammonia-N and MCP values of the 48 h fermentation fluid were analyzed using a microplate reader following the chemical methods described by Hristov et al. [17] and Makkar et al. [18], respectively.

The microbial DNA from the 48 h fermentation fluid samples was extracted using the E.Z.N.A.^®^ DNA Kit (Omega Bio-tek, Norcross, GA, USA), according to the manufacturer’s instructions. The quality and concentration of DNA were determined via 1.0% agarose gel electrophoresis and a NanoDrop2000 spectrophotometer (Thermo Scientific, Waltham, MA, USA), and DNA samples were kept at −80 °C prior to further use. The hypervariable region 27F_1492R of the bacterial 16S rRNA gene was amplified with the primer pair 27F (5′-AGRGTTYGATYMTGGCTCAG-3′) and 1492R (5′-RGYTACCTTGTTACGACTT-3′) using a T100 Thermal Cycler PCR thermocycler (BIO-RAD, USA). The PCR reaction mixture included 10 μL 2 × Pro Taq, 0.8 μL of each primer (5 μM), 10 ng/μL of template DNA, and ddH_2_O to a final volume of 20 µL. The PCR amplification cycling conditions were as follows: initial denaturation at 95 °C for 3 min; followed by 27 cycles of denaturing at 95 °C for 30 s, annealing at 55 °C for 30 s, and extension at 72 °C for 45 s; and single extension at 72 °C for 10 min, ending at 10 °C. The PCR product was extracted from 2% agarose gel and purified using the PCR Clean-Up Kit (YuHua, Shanghai, China), according to the manufacturer’s instructions, and quantified using Qubit 4.0 (Thermo Fisher Scientific, Waltham, MA, USA). Purified amplicons were pooled in equimolar amounts and paired-end sequenced on an Illumina Nextseq2000 platform (Illumina, San Diego, CA, USA), according to standard protocols, by Majorbio Bio-Pharm Technology Co., Ltd. (Shanghai, China). High-fidelity (HiFi) reads were obtained from the subreads, generated using circular consensus sequencing via SMRT Link v11.0.

HiFi reads were barcode-identified and length-filtered, and sequences with a length of <1000 or >1800 bp were removed. The optimized HiFi reads were clustered into operational taxonomic units (OTUs) using UPARSE 7.1 with a sequence similarity level of 97%. The most abundant sequence for each OTU was selected as a representative sequence, and the OTU table was manually filtered. To minimize the effects of sequencing depth on the alpha and beta diversity measures, the number of 16S rRNA gene sequences from each sample were rarefied to 6000, which still yielded an average Good’s coverage of 99.09%.

The richness and diversity of rumen microbial communities among the 4 groups were analyzed using the α diversity index (Sobs, Chao1, Ace, Simpson, coverage, and Shannon). Linear discriminant analysis (LDA) effect size (LEfSe) with an LDA score > 2 was used to determine specific bacteria among the 4 groups. The microbial data were analyzed using Majorbio Cloud (https://www.majorbio.com/; accessed on 10 October 2025).

### 2.5. Statistical Analysis

All statistical analyses were performed using the SAS software package (v9.4, SAS Institute Inc., Cary, NC, USA). Data were analyzed via one-way ANOVA using the MIXED procedure of SAS. The statistical model that describes the analysis is as follows:

Y_ij_ = µ + FP_i_ + Time_j_ + Run_k_ + e_ijk_

where Y_ij_ denotes the dependent variable (e.g., nutrient digestibility, gas production, fermentation traits) for treatment I, time j and run k; µ is the overall mean; FP_i_ is the fixed effect of the enzyme dose (CON, TRE1, TRE2, TRE3), used to test the primary hypothesis regarding the enzyme dose; Time_j_ is fixed effect of the incubation time (6, 12, 24, 48 h). Run_k_ denotes the random effect of the experimental run (Run 1 and Run 2), in order to account for the block effect and variability between the two experimental runs; and e_ij_ is the residual experimental error. The data of gas production and nutrient digestibility was analyzed at each fermentation times. Differences were declared significant at *p* < 0.05, and a trend toward significance was considered at 0.05 ≤ *p* < 0.10.

Spearmen’s rank correlations were calculated to measure the relationships between the relative abundances of the top 30 genera of rumen bacteria and fermentation parameters (i.e., gas production, DMD, NDFD, ADFD, and fermentation parameters) at 48 h, using the “corrplot” package in R (Version 3.6.3).

## 3. Results

### 3.1. Gas Production and In Vitro Nutrient Digestibility

As incubation time increased, the gas production and nutrient digestibility parameters were increased (*p* < 0.05). As shown in Table 2, there was no significant difference in gas production among the four groups at 3 h (*p* > 0.05); however, gas production was increased in the TRE1 and TRE3 groups compared to CON at 6, 9, 12, 24, and 48 h (*p* < 0.05). In addition, gas production did not differ between TRE2 and CON at 6, 9, and 12 h (*p* > 0.05), but was improved in TRE2 at 24 and 48 h (*p* < 0.05). Furthermore, DMD was the highest in the TRE3 group and the lowest in the CON group at 6, 12, 24, and 48 h (*p* < 0.05); while NDFD was higher in the TRE1 group and the lowest in the CON group at 6 h, but the highest in the TRE3 group and the lowest in the CON group at 12, 24, and 48 h (*p* < 0.05). There were no significant differences in ADFD among the four groups at 6 and 12 h; however, it was improved in the TRE2 and TRE3 groups compared to CON at 24 h, and improved in the TRE1, TRE2, and TRE3 groups compared to CON group at 48 h.

### 3.2. In Vitro Fermentation Parameters in the Fermentation Fluid at 48 h

As shown in Table 3, the pH; ammonia-N, butyrate, isobutyrate, valerate, and isovalerate concentrations; and the ratio of acetate to propionate in fermentation fluid at 48 h was not affected by supplementation with cellulases and pectinases. In contrast, the concentrations of MCP, TVFAs, acetate, and propionate were the highest in the TRE3 group and the lowest in the CON group (*p* < 0.05).

### 3.3. Bacterial Community Composition in the Fermentation Fluid at 48 h

**Figure 1:**
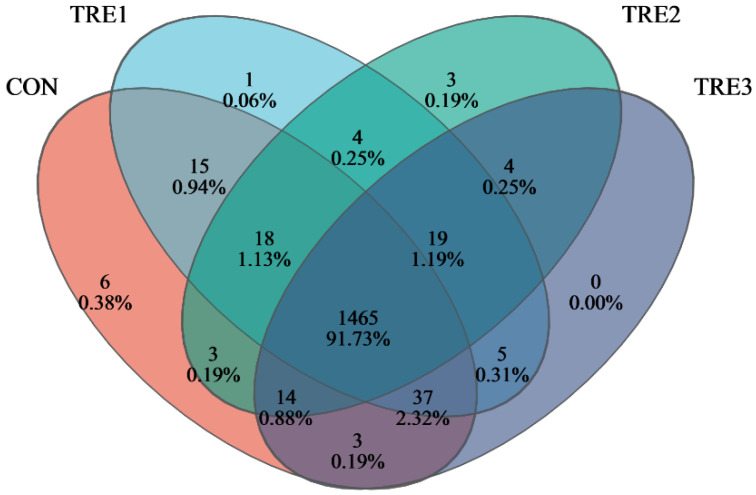
Flower plot showing different and similar OTU in goats when Macadamia integrifolia husk were used as substrates in vitro different level of exogenous enzymes.

**Figure 2:**
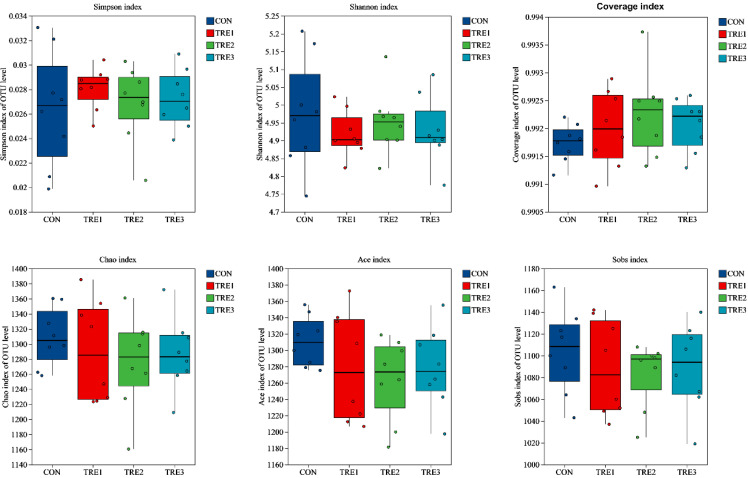
Alpha diversity indices of the rumen microbiota at the OTU level across Macadamia integrifolia husk as substrate in vitro with different level of exogenous enzymes. CON, control group, Macadamia integrifolia husk with no cellulases and pectinase; TRE1, treatment 1, Macadamia integrifolia husk with 0.5 g/kg cellulases and 0.5 g/kg pectinase; TRE2, treatment 2, Macadamia integrifolia husk with 1.0 g/kg cellulases and 0.5 g/kg pectinase; TRE3, treatment 3, Macadamia integrifolia husk with 1.5 g/kg cellulases and 0.5 g/kg pectinase.

A total of 1597 operational taxonomic units (OTUs) were generated from the 32 samples (Figure 1), of which 1465 OTUs were shared among the four groups. The numbers of OTUs specific to the CON, TRE1, TRE2, and TRE3 groups were 6, 1, 3, and 0, respectively. There were no significant differences in the Simpson, Shannon, coverage, Chao, Ace, and Sobs indices among the four groups (Figure 2).

**Figure 3:**
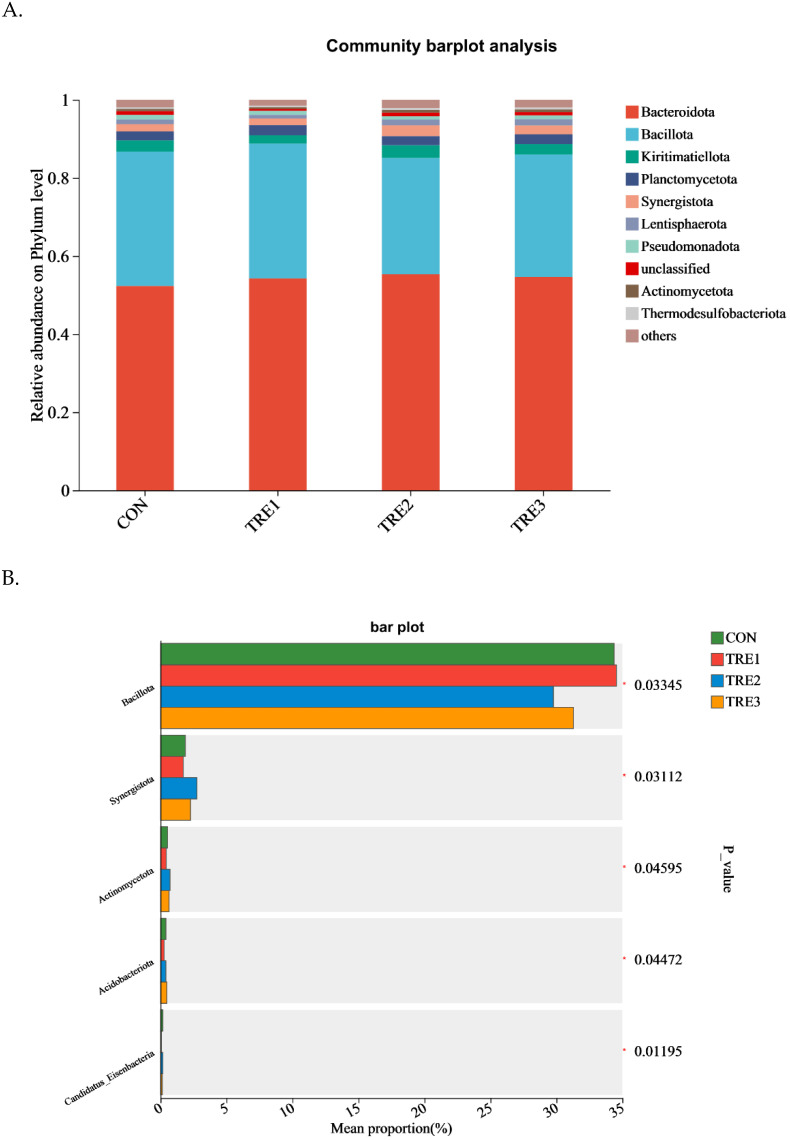
Bacterial relative abundances of the top 10 phylum level in goats when used as Macadamia integrifolia husk as substrate in vitro with different level of exogenous enzymes (**A**,**B**). CON, control group, Macadamia integrifolia husk with no cellulases and pectinase; TRE1, treatment 1, Macadamia integrifolia husk with 0.5 g/kg cellulases and 0.5 g/kg pectinase; TRE2, treatment 2, Macadamia integrifolia husk with 1.0 g/kg cellulases and 0.5 g/kg pectinase; TRE3, treatment 3, Macadamia integrifolia husk with 1.5 g/kg cellulases and 0.5 g/kg pectinase. * *p* < 0.05 according to the correlation coefficient.

A total of 28 phyla were identified among the four groups (Figure 3A; Supplementary Table S1). Bacteroidota was the top *phylum*, comprising approximately 52.3%, 54.2%, 55.3%, and 54.6% of the total bacteria in the CON, TRE1, TRE2, and TRE3 groups, respectively. Bacillota was the second most abundant phylum, comprising approximately 34.4%, 34.5%, 29.8%, and 31.3% of the total bacteria in the CON, TRE1, TRE2, and TRE3 groups, respectively. The Bacillota was the highest in the TRE1 group and the lowest in the TRE2 group (*p* < 0.05; Figure 3B), whereas Synergistota and Actinomycetota were the lowest in the TRE group and the highest in the TRE2 group (*p* < 0.05). The abundance of Acidobacteriota was the highest in the TRE3 group and the lowest in the TRE1 group (*p* < 0.05).

**Figure 4:**
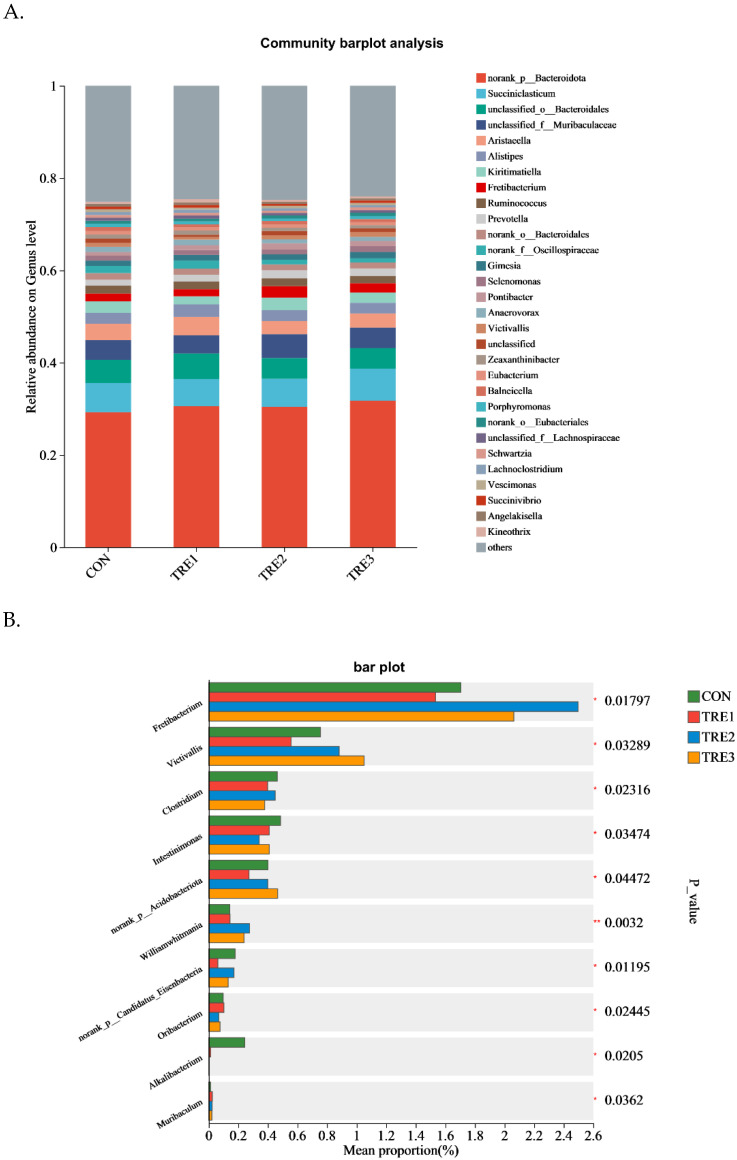
Bacterial relative abundances of the top 30 genus in goats when used as Macadamia integrifolia husk as substrate in vitro with different levels of exogenous enzymes (**A**,**B**). CON, control group, Macadamia integrifolia husk with no cellulases and pectinase; TRE1, treatment 1, Macadamia integrifolia husk with 0.5 g/kg cellulases and 0.5 g/kg pectinase; TRE2, treatment 2, Macadamia integrifolia husk with 1.0 g/kg cellulases and 0.5 g/kg pectinase; TRE3, treatment 3, Macadamia integrifolia husk with 1.5 g/kg cellulases and 0.5 g/kg pectinase. * *p* < 0.05 and ** *p* < 0.01 according to the correlation coefficient.

A total of 384 genera were identified among the four groups (Figure 4A, Supplementary Table S2). The norank_p_Bacteroidota was the top genus, comprising approximately 29.3%, 30.6%, 30.4%, and 31.7% of the total bacteria in the CON, TRE1, TRE2, and TRE3 groups, respectively. Succiniclasticum was the second most abundant genus, comprising approximately 6.3%, 5.8%, 6.1%, and 7.0% of the total bacteria in the CON, TRE1, TRE2, and TRE3 groups, respectively.

**Figure 5:**
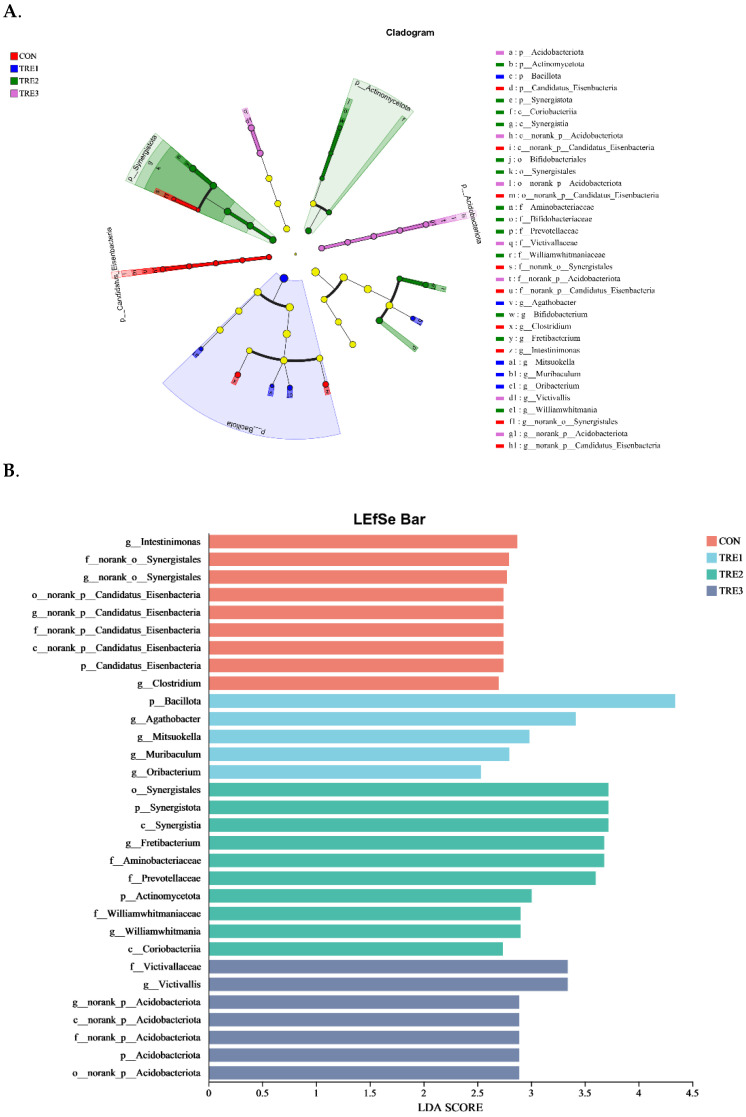
The significantly differential bacterial based on the linear discriminant analysis effect size (LEfSe) cladogram (**A**) and LDA score (**B**). CON, control group, Macadamia integrifolia husk with no cellulases and pectinase; TRE1, treatment 1, Macadamia integrifolia husk with 0.5 g/kg cellulases and 0.5 g/kg pectinase; TRE2, treatment 2, Macadamia integrifolia husk with 1.0 g/kg cellulases and 0.5 g/kg pectinase; TRE3, treatment 3, Macadamia integrifolia husk with 1.5 g/kg cellulases and 0.5 g/kg pectinase.

To identify the taxon distributions among these four groups, LEfSe analysis was performed, and biomarkers of fermentation fluid were assessed in the CON, TRE1, TRE2, and TRE3 groups, which revealed 8, 5, 10, and 7 biomarkers, respectively (Figure 5A,B).

### 3.4. In Vitro Rumen Fermentation Parameters Correlated with Bacterial Community Composition

**Figure 6:**
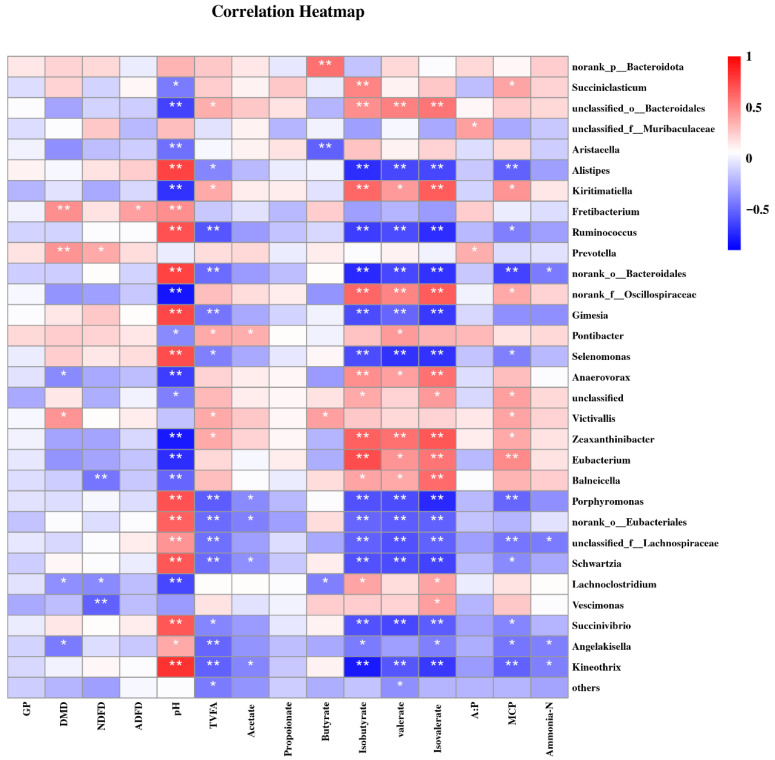
The correlations between the rumen fermentation parameters at 48 h and bacterial communities at the top 30 genus. GP, gas production; DMD, dry matter digestibility; NDFD, neutral detergent fiber digestibility; ADFD, acid detergent fiber digestibility; TVFA, total concentration of volatile fatty acid. A:P, the ratio of acetate to propionate; MCP, microbial protein. * *p* < 0.05 and ** *p* < 0.01 according to the correlation coefficient.

A total of 152 significant correlations (65 positive and 87 negative) between the relative abundances of the top 30 genera and in vitro fermentation indices were determined (Figure 6). The results of the present study show that the genus *Prevotella* was positively related with DMD, NDFD, and the ratio of acetate to propionate; *Anaerovorax* was positively related with isobutyrate, valerate, and isovalerate concentrations, but negatively related with DMD and pH; *Alistipes*, *Ruminococcus*, *Selenomonas*, and *Schwartzia* were positively related with pH, but negatively related with TVFA, isobutyrate, valerate, isovalerate, and MCP; and *Zeaxanthionibacter* and *Eubacterium* were negatively related to pH, but positively correlated with TVFA, isobutyrate, valerate, isovalerate, and MCP.

## 4. Discussion

For the present study, we evaluated the chemical composition of Macadamia integrifolia husk. The crude protein was 8.14%, higher than that in other common agri-industrial by-products—for example, corn stalk (5.90%) [19], soybean straw (3.67%) [20], cotton stalk (6.85%) [21], or wheat stalk (5.45%) [19]—but lower than that in almond skin (12.9%), hazelnut skin (10.2%), or pistachio skin (22.4%) [11].

Unfortunately, we ignored the plant bioactive compounds in Macadamia integrifolia husk in the present study, such as polyphenols and unsaturated fatty acids, which could decrease methane production and modulate rumen fermentation processes; namely, rumen biohydrogenation [11,22]. Hence, we believe that Macadamia integrifolia husk has huge potential as a feedstuff, and more scientific information concerning the use of Macadamia integrifolia husk as animal feedstuff is needed.

### 4.1. Effect of Macadamia Integrifolia Husk with Supplementary Cellulases and Pectinases on Gas Production and Nutrients Digestibility in Goats

In in vitro studies, gas production is regarded as a vital index reflecting the speed at which rumen microorganisms ferment substrate nutrients, with a higher gas production generally indicating a greater degree of substrate fermentation by the microorganisms [23,24]. In vivo studies have reported that exogenous enzymes could improve the nutritive value of agri-industrial by-products due to enhanced attachment by rumen microorganisms, consequently improving animal production performance [25,26]. In the present study, we found that gas production was increased when Macadamia integrifolia husk was supplemented with cellulases and pectinases, leading to improved dry matter digestibility, neutral detergent fiber digestibility, and acid detergent fiber digestibility. These results are in agreement with previous in vitro studies of fibrolytic enzymes in tropical forages on Santa Inês sheep [27], xylanase enzyme in wheat straw on Mongolian native goats [28], and multifunctional xylanase in wheat straw on beef cattle [29]. Pectin is a non-fiber carbohydrate which widely exists in agro-industrial by-products, making the use of pectinases necessary to improve their utilization. In a previous study, while all addition levels of pectinases increased dietary DMD, NDFD, and ADFD, the maximal level of degradability was obtained with the addition of pectinases at 600 IU/kg dry matter, leading to 11.44% improvements over the control [30].

### 4.2. Effect of Macadamia Integrifolia Husk with Supplementary Cellulases and Pectinases on Rumen Fermentation Parameters in Goats

Rumen fluid pH is a comprehensive index reflecting rumen fermentation and health status. The relative abundances of Bacteroidetes, Patescibacteria, and Proteobacteria have been reported to reduce when the pH decreases by 0.5 units [31]. In the present study, we found that the pH of the fermentation fluid ranged between 6.37 and 6.39, which is within the normal ruminal pH range [31]. Ammonia is a vital nitrogen source for many rumen microbes to synthesize microbial proteins. The concentration of ammonia-N ranged between 30.57 and 32.61 mg/100 mL, which is above the minimum concentration of ammonia-N in an in vitro study (5 g/100 mL) [32]. In addition, our results indicated that the ammonia-N did not significantly differ among the four groups, in agreement with a meta-analysis reporting that the ruminal ammonia nitrogen concentration is not altered by enzymes [33]. In addition, the increased concentration of MCP could also explain the ammonia-N utilized by microbes. The MCP synthesized in the rumen supplies more than half of all the metabolizable protein required by ruminants [34]. A previous study showed that the MCP content in the 500 mg/kg non-starch polysaccharide enzymes group showed an increasing trend in goats [35], while another study reported that the ruminal ammonia-N concentration tended to be higher with recombinant fibrolytic enzymes as compared to control in heifers [36]. The difference in the MCP values could be explained by the dose and composition of the enzymes, as well as the differences between in vitro and in vivo studies.

Ran et al. (2019) reported that the concentrations of TVFA and molar proportion of individual VFAs did not differ between the control treatment and one including recombinant fibrolytic enzymes [36]. A previous in vivo study reported that feeding Pektofoetidin G3x (mostly pectinase and cellulase) to adult wethers and weaned lambs altered the composition of VFAs; for example, propionate increased while the ratio of acetate to propionate decreased [37]. In contrast, Simon et al. reported that dietary supplementation with 0.5 g enzyme blend + 0.5 g amylase per kg of DM led to a higher concentration of acetate than that in the other treatment groups, which were equal to each other. Treatment with 0.5 g of amylase per kg of DM in the diet and treatment with 0.5 g enzyme blend + 0.5 g amylase per kg of DM in the diet led to a higher concentration of propionate than in the CON group. The concentration of butyrate had a strong tendency to be higher in the treatment with 0.5 g enzyme blend + 0.5 g amylase per kg of DM in the diet, compared to the CON group. Meanwhile, no treatment effects for isovalerate and valerate concentrations were observed between groups [38]. Moreover, an in vitro study has reported increased acetate and butyrate and decreased propionate molar proportions in steers, lactating dairy cows, and ruminal-simulating continuous cultures with the dietary addition of α-amylase [39]. In the present study, we found that the concentrations of TVFAs, acetate, and propionate were higher in TRE3 group than in the CON group. These results partly agree with those of a previous in vivo study conducted in heifers and feedlot cattle. Moreover, the improvements in DMD and NDFD could also explain the high yields of TVFA and acetate.

### 4.3. Effect of Macadamia Integrifolia Husk with Supplementary Cellulases and Pectinases on Bacterial Community Composition in Goats

The ruminal microorganisms contribute to the digestion of fibrous plant materials and the extraction of essential nutrients. These microbes are adept at breaking down complex carbohydrates, such as cellulose and hemicellulose, into simpler sugars and short-chain fatty acids through fermentation processes. There was no significant difference in the bacterial richness and diversity in dairy cows between the CON group and those fed a combination of fibrolytic and amylolytic enzymes [40]. However, a previous study reported that the Chao and Ace indices of the experimental group were significantly lower than those of the control group, and the Shannon and Simpson indices were lower than those of the control group in Tan sheep [41]. In the present study, we found that the Simpson, Shannon, Coverage, Chao, Sobs, and Ace indices were not affected by the different levels of enzymes, indicating that the fermentation fluid community structure remained relatively stable.

As reported in previous studies, Bacteroidota and Bacillota are the dominant phyla in goats [42] and Hu sheep [43], in agreement with our results. Most Synergistota are asaccharolytic, but all share the ability to ferment amino acids [44]. The relative abundance of Synergistota was higher in the TRE2 and TRE3 groups compared to CON, which could explain the increase in MCP concentration in these treatment groups. A previous study reported that Acidobacteriota could enhance the digestibility of nutrients, as they play an especially vital role in the breakdown of organic matter. Interestingly, in the present study, we found that the relative abundance of Acidobacteriota was the highest in TRE3 group, potentially resulting in an improvement in organic matter digestibility when supplemented with exogenous enzymes. Unfortunately, the digestibility of organic matter was not measured in the present study, and this hypothesis should be verified further research.

Li et al. reported that *Escherichia-Shigella* and *Streptococcus* were the dominant genera in an in vitro study on Taihang White cashmere goats [45]. Another study showed that the Prevotella 1 was the dominant genus in an in vitro study on Crossbreed Boer female goats [46]. In the present study, we found that the dominant genera were *norank_p_Bacteroidota*, followed by *Succiniclasticum*. This difference between the reported genera could be explained by the rumen fluid donor, the animal species, and the chemical composition of the animals’ feed. *Vicivalli*, belonging to phylum Lentisphaerae, is regarded as a cellobiose-degrading organism isolated from human feces [47]. Moreover, it has been positively associated with high nitrogen retention and average daily gain in beef cattle [48]. In the present study, we found that the relative abundance of *Victivallis* was the highest in the TRE3 group and was positively associated with MCP concentrations, which could explain the improvement of acetate concentrations and MCP concentration, thus providing energy and protein for their host. A positive correlation has been reported between the genus *Fretibacterium* and polyunsaturated fatty acids and conjugated linoleic acids [49]. In the present study, as the substrate of Macadamia integrifolia husk was enriched in fatty acids, this may have improved the relative abundance of *Fretibacterium*. Additionally, *Fretibacterium* was found to be positively associated with DMD. Our results showed positive correlations between *Prevotella* and DMD and NDFD; in agreement with *Prevotella*’s known role as the major pectinolytic bacteria in the rumen [50]. The genus Anaerovorax was associated with high nitrogen utilization in beef cattle [48]. In the present study, we found that Anaerovorax was positively associated with the isobutyrate, valerate, and isovalerate concentrations, all of which are precursors for the synthesis of MCP.

## 5. Conclusions

In the present study, using Macadamia integrifolia husk as a substrate, we found that gas production, DMD, NDFD, and ADFD were higher in the cellulases- and pectinases-treated groups compared to controls. In addition, the concentrations of MCP, TVFAs, acetate, and propionate were also improved in the cellulases- and pectinases-treated groups. These results provide new insights regarding the use of Macadamia integrifolia husk as a feedstuff in goats; in particular, supplementation with cellulases and pectinases could effectively improve its utilization. The optimal supplementary levels of cellulases and pectinases were determined as 1.5 g/kg and 0.5 g/kg, respectively. In future research, an in vivo study should be carried out to provide more information on the quality of Macadamia integrifolia husk and its effects on growth performance when used as a feedstuff.

## Figures and Tables

**Table 1:** Chemical composition of the Macadamia integrifolia husk.

Items	Chemical Composition, %
Dry matter	93.7
Crude protein	8.14
Organic matter	89.9
Ether extract	8.53
Neutral detergent fiber	41.3
Acid detergent fiber	29.6

**Table 2:** Effect of different levels of exogenous enzymes in the Macadamia integrifolia husk on gas production and nutrients digestibilities in vitro.

**Items**	**Times**	**CON**	**TRE1**	**TRE2**	**TRE3**	**SEM**	***p***
Gas production, mL/0.4 g of dry matter	3 h	33.33	35.03	33.70	34.88	0.354	0.237
	6 h	51.05 ^a^	55.65 ^b^	52.26 ^a^	55.83 ^b^	0.510	<0.001
	9 h	66.03 ^ab^	69.80 ^b^	65.21 ^a^	68.49 ^ab^	0.578	0.01
	12 h	75.36 ^a^	80.64 ^b^	77.06 ^ab^	81.45 ^b^	0.805	0.014
	24 h	97.28 ^a^	114.50 ^c^	105.53 ^b^	111.18 ^c^	1.292	<0.001
	48 h	126.53 ^a^	148.68 ^c^	134.83 ^b^	145.38 ^c^	1.683	<0.001
Dry matter digestibility, %	6 h	29.20 ^a^	30.64 ^ab^	32.16 ^b^	33.14 ^b^	0.453	<0.01
	12 h	33.17 ^a^	37.07 ^b^	36.58 ^b^	37.72 ^b^	0.428	<0.001
	24 h	40.64 ^a^	42.95 ^a^	42.23 ^a^	44.99 ^b^	1.137	0.048
	48 h	45.60 ^a^	47.47 ^a^	51.41 ^b^	52.75 ^b^	0.579	<0.001
Neutral detergent fiber digestibility, %	6 h	21.69 ^a^	23.38 ^b^	22.46 ^ab^	22.76 ^ab^	0.343	0.042
	12 h	28.94 ^a^	29.81 ^a^	30.02 ^ab^	31.44 ^b^	0.759	0.044
	24 h	37.02 ^a^	39.85 ^b^	37.71 ^a^	41.26 ^b^	0.457	<0.001
	48 h	41.78 ^a^	45.12 ^b^	44.82 ^b^	45.92 ^b^	0.346	<0.001
Acid detergent fiber digestibility, %	6 h	13.93	14.77	14.44	13.87	0.235	0.490
	12 h	21.09	20.18	21.65	20.70	0.365	0.570
	24 h	30.83 ^a^	30.44 ^a^	32.55 ^b^	32.35 ^b^	0.374	0.040
	48 h	33.71 ^a^	36.32 ^b^	36.40 ^b^	37.15 ^b^	0.290	<0.001

SEM, standard error of the mean. Different superscript letters within a column represent a significant difference among the 4 groups. CON, control group, Macadamia integrifolia husk with no cellulases and pectinase; TRE1, treatment 1, Macadamia integrifolia husk with 0.5 g/kg cellulases and 0.5 g/kg pectinase; TRE2, treatment 2, Macadamia integrifolia husk with 1.0 g/kg cellulases and 0.5 g/kg pectinase; TRE3, treatment 3, Macadamia integrifolia husk with 1.5 g/kg cellulases and 0.5 g/kg pectinase.

**Table 3:** Effect of different level of exogenous enzymes to the Macadamia integrifolia husk on rumen fermentation parameters at 48 h in vitro.

Items	CON	TRE1	TRE2	TRE3	SEM	*p*
pH	6.38	6.37	6.38	6.39	0.04	0.999
Ammonia-N, mg/100 mL	30.57	32.61	31.78	32.41	0.35	0.150
MCP, mg/100 mL	19.32 ^a^	20.21 ^ab^	20.20 ^ab^	21.17 ^b^	0.222	0.024
TVFAs, mmol/L	45.21 ^a^	47.14 ^ab^	46.31 ^ab^	48.33 ^c^	0.290	<0.001
Acetate, mmol/L	31.50 ^a^	32.73 ^bc^	32.33 ^ab^	33.66 ^c^	0.183	<0.001
Propionate, mmol/L	8.01 ^a^	8.31 ^bc^	8.12 ^ab^	8.44 ^c^	0.040	<0.001
Butyrate, mmol/L	0.40	0.42	0.41	0.43	0.014	0.826
Iso-butyrate, mmol/L	2.40	2.54	2.46	2.61	0.061	0.645
Valerate, mmol/L	1.94	2.09	1.99	2.13	0.082	0.855
Iso-valerate, mmol/L	0.86	1.04	1.00	1.07	0.031	0.706
Acetate–Propionate	3.94	3.94	3.98	3.99	0.017	0.541

SEM, standard error of the mean; MCP, microbial protein; TVFA, total volatile fatty acids. Different superscript letters within a column represent a significant difference among the 4 groups. CON, control group, Macadamia integrifolia husk with no cellulases and pectinase; TRE1, treatment 1, Macadamia integrifolia husk with 0.5 g/kg cellulases and 0.5 g/kg pectinase; TRE2, treatment 2, Macadamia integrifolia husk with 1.0 g/kg cellulases and 0.5 g/kg pectinase; TRE3, treatment 3, Macadamia integrifolia husk with 1.5 g/kg cellulases and 0.5 g/kg pectinase.

## Data Availability

The datasets presented in this study are available from the authors on reasonable request.

## Supplementary Materials

The following supporting information can be downloaded at: https://www.mdpi.com/article/10.3390/ani15223337/s1, Table S1: Bacterial relative abundances of the top 10 phylum level in goats when used as Macadamia integrifolia husk as substrate in vitro with different level of exogenous enzymes; Table S2: Bacterial relative abundances of the top 30 genus in goats when used as Macadamia integrifolia husk as substrate in vitro with different level of exogenous enzymes.

## References

[1] Tu X.H., Wu B.F., Xie Y., Xu S.L., Wu Z.Y., Lv X., Wei F., Du L.Q., Chen H. (2021). A comprehensive study of raw and roasted macadamia nuts: Lipid profile, physicochemical, nutritional, and sensory properties. Food Sci. Nutr..

[2] Guo Q., Barkla B.J., Barker R., Liu L. (2025). Genotype-Driven proteomic diversity in macadamia nuts: Implications for allergenicity, nutritional quality, and breeding strategies. J. Agric. Food Chem..

[3] Shuai X., Dai T., Chen M., Liang R., Du L., Chen J., Liu C. (2021). Comparative Study of Chemical Compositions and Antioxidant Capacities of Oils Obtained from 15 Macadamia (*Macadamia integrifolia*) Cultivars in China. Foods.

[4] Yang F., Tang J., Fu X.M., Tang S.P., Yang H.X., Dong M.C., Luo X.P. (2025). Overview and prospect of production and marketing situation of macadamia in China. Fruit. Trees S. China.

[5] Ahmed M.F., Popovich D.G., Whitby C.P., Rashidinejad A. (2024). Phenolic compounds from macadamia husk: An updated focused review of extraction methodologies and antioxidant activities. Food Bioprod. Process.

[6] Yang K., Qing Y., Yu Q., Tang X., Chen G., Fang R., Liu H. (2021). By-Product feeds: Current understanding and future perspectives. Agriculture.

[7] Haider M.W., Abbas S.M., Saeed M.A., Farooq U., Waseem M., Adil M., Tutu C. (2025). Osei. Environmental and nutritional value of fruit and vegetable peels as animal feed: A comprehensive review. Anim. Res. One Health.

[8] Gao Q., Liu H., Wang Z., Lan X., An J., Shen W., Wan F. (2023). Recent advances in feed and nutrition of beef cattle in China—A review. Anim. Biosci..

[9] Sakita G.Z., Bompadre T.F.V., Dineshkumar D., Lima P.M.T., Abdalla Filho A.L., Campioni T.S., de Oliva Neto P., Bremer Neto H., Louvandini H., Abdalla A.L. (2020). Fibrolytic enzymes improving in vitro rumen degradability of tropical forages. J. Anim. Physiol. Anim. Nutr..

[10] Coblentz W.K., Akins M.S. (2018). Silage review: Recent advances and future technologies for baled silages. J. Dairy Sci..

[11] Musati M., Hervás G., Natalello A., Toral P.G., Luciano G., Priolo A., Frutos P. (2024). Could we partially replace maize with nut skins for more sustainable sheep diets? In vitro ruminal fermentation and biohydrogenation. Anim. Feed. Sci. Technol..

[12] Musati M., Frutos P., Bertino A., Hervás G., Luciano G., Forte C., Priolo A., Lanza M., Bella M., Biondi L. (2024). Dietary combination of linseed and hazelnut skin as a sustainable strategy to enrich lamb with health promoting fatty acids. Sci. Rep..

[13] Liu H., Li Z., Pei C., Degen A., Hao L., Cao X., Liu H., Zhou J., Long R. (2022). A comparison between yaks and Qaidam cattle in in vitro rumen fermentation, methane emission, and bacterial community composition with poor quality substrate. Anim. Feed. Sci. Technol..

[14] AOAC (2012). Official Methods of Analysis of the Association of Official Analytical Chemists.

[15] Van Soest P.J., Robertson J.B., Lewis B.A. (1991). Methods for dietary fiber, neutral detergent fiber, and nonstarch polysaccharides in relation to animal nutrition. J. Dairy. Sci..

[16] Robertson J.B., Van Soest P.J., James W.P., Theander O. (1981). The detergent system of analysis and its application to human foods. The Analysis of Dietary Fibres in Food.

[17] Hristov A.N., Ivan M., Rode L.M., McAllister T.A. (2001). Fermentation characteristics and ruminal ciliate protozoal population in cattle fed medium- or high-concentrate barley-based diets. J. Anim. Sci..

[18] Makkar H., Sharma O., Dawra R., Negi S. (1982). Simple determination of microbial protein in rumen liquor. J. Dairy Sci..

[19] Wang B., Mao S.Y., Yang H.J., Wu Y.M., Wang J.K., Li S.L., Shen Z.M., Liu J.X. (2014). Effects of alfalfa and cereal straw as a forage source on nutrient digestibility and lactation performance in lactating dairy cows. J. Dairy. Sci..

[20] Wei M., Cui Z., Li J., Yan P. (2018). Estimation of metabolisable energy and net energy of rice straw and wheat straw for beef cattle by indirect calorimetry. Arch. Anim. Nutr..

[21] Shen R.R., Sun X.Y., Liu B., Li Y.Q., Gao Y.X., Li J.G., Cao Y.F., Li Q.F. (2019). Effects of different compound microorganism preparations on fermentation quality, nutritional components and rumen degradation rate of mixed silage of potato pulp and soybean straw. Chin. J. Anim. Nutr..

[22] Zhang Z.J., Guo T.J., Zhao J., Sang D.J., Shi Y., Cui J.W. (2018). Effects of steam explosion and fermentation after steam explosion on nutrient value of cotton stalk. Chin. J. Anim. Nutr..

[23] Yang Z., Zheng Y., Liu S., Xie T., Wang Q., Wang Z., Li S., Wang W. (2024). Rumen metagenome reveals the mechanism of mitigation methane emissions by unsaturated fatty acid while maintaining the performance of dairy cows. Anim. Nutr..

[24] Pashaei S., Razmazar V., Mirshekar R. (2010). Gas Production: A Proposed in vitro Method to Estimate the Extent of Digestion of a Feedstuff in the Rumen. J. Biol. Sci..

[25] Christodoulou C., Kliem K.E., Auffret M.D., Humphries D.J., Newbold J.R., Davison N., Crompton L., Dhanoa M.S., Smith L.G., Stergiadis S. (2025). In vitro rumen degradation, fermentation, and methane production of four agro-industrial protein-rich co-products, compared with soyabean meal. Anim. Feed. Sci. Technol..

[26] Bugoni M., Takiya C.S., Grigoletto N.T.S., Vittorazzi Júnior P.C., Nunes A.T., Chesini R.G., da Silva G.G., Durman T., Pettigrew J.E., Rennó F.P. (2023). Feeding amylolytic and proteolytic exogenous enzymes: Effects on nutrient digestibility, ruminal fermentation, and performance in dairy cows. J. Dairy Sci..

[27] Liu Z., Li W., Zhao C., Zhang Y., Li Y., Wang L., Li X., Yao J., Pellikaan W.F., Cao Y. (2024). Effects of fibrolytic and amylolytic compound enzyme preparation on rumen fermentation, serum parameters and production performance in primiparous early-lactation dairy cows. J. Dairy Res..

[28] Togtokhbayar N., Cerrillo M.A., Rodríguez G.B., Elghandour M.M., Salem A.Z., Urankhaich C., Jigjidpurev S., Odongo N.E., Kholif A.E. (2015). Effect of exogenous xylanase on rumen in vitro gas production and degradability of wheat straw. Anim. Sci. J..

[29] Zhang M., Qiu Q., Zhao X., Ouyang K., Liu C. (2024). Characterization of novel multifunctional xylanase from rumen metagenome and its effects on in vitro microbial fermentation of wheat straw. Fermentation.

[30] Azzaz H.H., Murad H.A., Hassaan Noha A., Fahmy M. (2020). Pectinase Production Optimization for Improving Dairy Animal’s Diets Degradation. Int. J. Dairy Sci..

[31] Li M.M., White R.R., Guan L.L., Harthan L., Hanigan M.D. (2021). Metatranscriptomic analyses reveal ruminal pH regulates fiber degradation and fermentation by shifting the microbial community and gene expression of carbohydrate-active enzymes. Anim. Microbiome.

[32] Dewhurst R.J., Newbold J.R. (2022). Effect of ammonia concentration on rumen microbial protein production in vitro. Br. J. Nutr..

[33] Ferreira I.M., Mantovani H.C., Vedovatto M., Cardoso A.S., Rodrigues A.A., Homem B.G.C., de Abreu M.J.I., Rodrigues A.N., Cursino Batista L.H., de Oliveira J.S. (2025). Impact of dietary exogenous feed enzymes on performance, nutrient digestibility, and ruminal fermentation parameters in beef cattle: A meta-analysis. Animal.

[34] NASEM (The National Academies of Sciences, Engineering, and Medicine) (2016). Nutrient Requirements of Beef Cattle.

[35] Tan Z., Wang L., Wang Z., Xue B., Hu R., Peng Q.H., Xiao J.X. (2025). Supplementing NSP enzymes in high concentrate diets can prevent foamy rumen bloat in goats. Sci. Rep..

[36] Ran T., Saleem A.M., Shen Y., Ribeiro G.O., Beauchemin K.A., Tsang A., Yang W., McAllister T.A. (2019). Effects of a recombinant fibrolytic enzyme on fiber digestion, ruminal fermentation, nitrogen balance, and total tract digestibility of heifers fed a high forage diet1. J. Anim. Sci..

[37] Baran M., Kmet V. (1987). Effect of pectinase on rumen fermentation in sheep and lambs. Arch. Anim. Nutr..

[38] Simon A.L., Copetti P.M., Lago R.V.P., Vitt M.G., Nascimento A.L., Silva L.E.L.E., Wagner R., Klein B., Martins C.S., Kozloski G.V. (2023). Inclusion of exogenous enzymes in feedlot cattle diets: Impacts on physiology, rumen fermentation, digestibility and fatty acid profile in rumen and meat. Biotechnol. Rep.

[39] Tricarico J.M., Johnston J.D., Dawson K.A., Hanson K.C., Mcleod K.R., Harmon D.L. (2005). The effects of an Aspergillus oryzae extract containing alpha-amylase activity on ruminal fermentation and milk production in lactating Holstein cows. Anim. Sci..

[40] Liu Z.K., Li Y., Zhao C.C., Liu Z.J., Wang L.M., Li X.Y., Pellikaan W.F., Yao J.H., Cao Y.C. (2022). Effects of a combination of fibrolytic and amylolytic enzymes on ruminal enzyme activities, bacterial diversity, blood profile and milk production in dairy cows. Animal.

[41] Jiang B., Wang T., Zhou Y., Li F. (2020). Effects of enzyme + bacteria treatment on growth performance, rumen bacterial diversity, KEGG pathways, and the CAZy spectrum of Tan sheep. Bioengineered.

[42] Xu J., Chen X., Ren J., Xu J., Zhang L., Yan F., Liu T., Zhang G., Huws S.A., Yao J. (2025). Multi-omics insights into microbiome-rumen epithelium interaction mechanisms underlying subacute rumen acidosis tolerance in dairy goats. Genome Biol..

[43] Zhao W., Abdelsattar M.M., Wang X., Zhang N., Chai J. (2023). In Vitro Modulation of Rumen Fermentation by Microbiota from the Recombination of Rumen Fluid and Solid Phases. Microbiol. Spectr..

[44] McSweeney C.S., Halliday M., Mackie R.I. (2025). Rumen Synergistota: New insights into their role in mimosine and fluoroacetate toxicity of ruminant livestock. Appl. Environ. Microbiol..

[45] Li J., Yan H., Chen J., Duan C., Guo Y., Liu Y., Zhang Y., Ji S. (2022). Correlation of ruminal fermentation parameters and rumen bacterial community by comparing those of the goat, sheep, and cow in vitro. Fermentation.

[46] Yu J., Cai L., Zhang J., Yang A., Wang Y., Zhang L., Guan L.L., Qi D. (2020). Effects of thymol supplementation on goat rumen fermentation and rumen microbiota in vitro. Microorganisms.

[47] Zoetendal E.G., Plugge C.M., Akkermans A.D.L., De Vos W.M. (2003). *Victivallis vadensis* gen. nov., sp. nov., a sugar-fermenting anaerobe from human faeces. Int. J. Syst. Evol. Microbiol..

[48] Gomes Carvalho Alves K.L., Granja-Salcedo Y.T., Messana J.D., de Souza V.C., Generoso Ganga M.J., Detogni Colovate P.H., Kishi L.T., Berchielli T.T. (2021). Rumen bacterial diversity in relation to nitrogen retention in beef cattle. Anaerobe.

[49] Mavrommatis A., Skliros D., Flemetakis E., Tsiplakou E. (2021). Changes in the rumen bacteriome structure and enzymatic activities of goats in response to dietary supplementation with *Schizochytrium* spp. Microorganisms.

[50] Nagaraja T.G., Millen D.D., Arrigoni M.D.B., Pacheco R.L. (2016). Microbiology of the rumen. Rumenology.

