# Supplementary material for: The Effects of Added Cellulases and Pectinases on Ruminal Fermentation Parameters and Bacterial Communities in Goats Supplemented with Macadamia Integrifolia Husks: An In Vitro Study
Source: Animals (Basel). 2025 Nov 19;15(22):3337. doi: 10.3390/ani15223337 (PMC12649204; doi:10.3390/ani15223337)
Supplement: Supplementary file 1 [file animals-15-03337-s001.zip › animals-3966789-supplementary.pdf]

**Supplementary Table S1** Bacterial relative abundances of the top 10 phylum level in goats when used as Macadamia integrifolia husk as substrate in vitro with different level of exogenous enzymes.

| OTU ID | CON | TRE1 | TRE2 | TRE3 |
|---|---|---|---|---|
| Bacteroidota | 0.5230 | 0.5424 | 0.5532 | 0.5463 |
| Bacillota | 0.3441 | 0.3453 | 0.2977 | 0.3134 |
| Kiritimatiellota | 0.0291 | 0.0210 | 0.0328 | 0.0268 |
| Planctomycetota | 0.0227 | 0.0263 | 0.0231 | 0.0252 |
| Synergistota | 0.0189 | 0.0169 | 0.0277 | 0.0226 |
| Lentisphaerota | 0.0120 | 0.0092 | 0.0147 | 0.0160 |
| Pseudomonadota | 0.0116 | 0.0103 | 0.0085 | 0.0100 |
| unclassified | 0.0098 | 0.0048 | 0.0095 | 0.0082 |
| Actinomycetota | 0.0054 | 0.0042 | 0.0071 | 0.0066 |
| Thermodesulfobacteriota | 0.0044 | 0.0043 | 0.0047 | 0.0052 |
| others | 0.0192 | 0.0152 | 0.0211 | 0.0199 |

CON, control group, Macadamia integrifolia husk with no cellulases and pectinase; TRE1, treat-ment 1, Macadamia integrifolia husk with 0.5 g/kg cellulases and 0.5 g/kg pectinase; TRE2, treat-ment 2, Macadamia integrifolia husk with 1.0 g/kg cellulases and 0.5 g/kg pectinase; TRE3, treat-ment 3, Macadamia integrifolia husk with 1.5 g/kg cellulases and 0.5 g/kg pectinase.

**Supplementary Table S2** Bacterial relative abundances of the top 30 genus in goats when used as Macadamia integrifolia husk as substrate in vitro with different level of exogenous enzymes.

| OTU ID | CON | TRE1 | TRE2 | TRE3 |
|---|---|---|---|---|
| *norank_p__Bacteroidota* | 0.2930 | 0.3061 | 0.3041 | 0.3174 |
| *Succiniclasticum* | 0.0631 | 0.0582 | 0.0613 | 0.0697 |
| *unclassified_o__Bacteroidales* | 0.0504 | 0.0557 | 0.0446 | 0.0446 |
| *unclassified_f__Muribaculaceae* | 0.0424 | 0.0395 | 0.0517 | 0.0443 |
| *Aristaeella* | 0.0352 | 0.0399 | 0.0286 | 0.0308 |
| *Alistipes* | 0.0242 | 0.0269 | 0.0239 | 0.0225 |
| *Kiritimatiella* | 0.0244 | 0.0176 | 0.0268 | 0.0222 |
| *Fretibacterium* | 0.0172 | 0.0150 | 0.0251 | 0.0205 |
| *Ruminococcus* | 0.0171 | 0.0171 | 0.0166 | 0.0161 |
| *Prevotella* | 0.0126 | 0.0142 | 0.0175 | 0.0161 |
| *norank_o__Bacteroidales* | 0.0146 | 0.0132 | 0.0129 | 0.0126 |
| *norank_f__Oscillospiraceae* | 0.0152 | 0.0177 | 0.0101 | 0.0094 |
| *Gimesia* | 0.0117 | 0.0125 | 0.0117 | 0.0137 |
| *Selenomonas* | 0.0104 | 0.0107 | 0.0098 | 0.0128 |
| *Pontibacter* | 0.0082 | 0.0102 | 0.0142 | 0.0109 |
| *Anaerovorax* | 0.0118 | 0.0119 | 0.0083 | 0.0088 |
| *Victivallis* | 0.0076 | 0.0057 | 0.0088 | 0.0103 |
| *unclassified* | 0.0098 | 0.0048 | 0.0095 | 0.0082 |
| *Zeaxanthinibacter* | 0.0088 | 0.0093 | 0.0071 | 0.0068 |
| *Eubacterium* | 0.0078 | 0.0072 | 0.0068 | 0.0068 |
| *Balneicella* | 0.0083 | 0.0059 | 0.0074 | 0.0063 |
| *Porphyromonas* | 0.0076 | 0.0066 | 0.0054 | 0.0070 |
| *norank_o__Eubacteriales* | 0.0063 | 0.0058 | 0.0063 | 0.0064 |
| *unclassified_f__Lachnospiraceae* | 0.0062 | 0.0066 | 0.0057 | 0.0057 |
| *Schwartzia* | 0.0061 | 0.0057 | 0.0051 | 0.0064 |
| *Lachnoclostridium* | 0.0063 | 0.0072 | 0.0051 | 0.0045 |
| *Vescimonas* | 0.0064 | 0.0042 | 0.0057 | 0.0051 |
| *Succinivibrio* | 0.0054 | 0.0059 | 0.0040 | 0.0058 |
| *Angelakisella* | 0.0065 | 0.0054 | 0.0043 | 0.0039 |
| *Kineothrix* | 0.0043 | 0.0072 | 0.0040 | 0.0042 |
| *others* | 0.2512 | 0.2458 | 0.2475 | 0.2399 |

CON, control group, Macadamia integrifolia husk with no cellulases and pectinase; TRE1, treat-ment 1, Macadamia integrifolia husk with 0.5 g/kg cellulases and 0.5 g/kg pectinase; TRE2, treat-ment 2, Macadamia integrifolia husk with 1.0 g/kg cellulases and 0.5 g/kg pectinase; TRE3, treat-ment 3, Macadamia integrifolia husk with 1.5 g/kg cellulases and 0.5 g/kg pectinase.
